# MeaningBERT: assessing meaning preservation between sentences

**DOI:** 10.3389/frai.2023.1223924

**Published:** 2023-09-22

**Authors:** David Beauchemin, Horacio Saggion, Richard Khoury

**Affiliations:** ^1^Group for Research in Artificial Intelligence of Laval University, Department of Computer Science and Software Engineering, Université Laval, Québec, QC, Canada; ^2^Large Scale Text Understanding System Lab, Natural Language Processing Group, Department of Information and Communication Technologies, Universitat Pompeu Fabra, Barcelona, Spain

**Keywords:** evaluation of text simplification systems, meaning preservation, automatic text simplification, lexical simplification, syntactic simplification, few-shot evaluation of text simplification systems

## Abstract

In the field of automatic text simplification, assessing whether or not the meaning of the original text has been preserved during simplification is of paramount importance. Metrics relying on n-gram overlap assessment may struggle to deal with simplifications which replace complex phrases with their simpler paraphrases. Current evaluation metrics for meaning preservation based on large language models (LLMs), such as BertScore in machine translation or QuestEval in summarization, have been proposed. However, none has a strong correlation with human judgment of meaning preservation. Moreover, such metrics have not been assessed in the context of text simplification research. In this study, we present a meta-evaluation of several metrics we apply to measure content similarity in text simplification. We also show that the metrics are unable to pass two trivial, inexpensive content preservation tests. Another contribution of this study is MeaningBERT (https://github.com/GRAAL-Research/MeaningBERT), a new trainable metric designed to assess meaning preservation between two sentences in text simplification, showing how it correlates with human judgment. To demonstrate its quality and versatility, we will also present a compilation of datasets used to assess meaning preservation and benchmark our study against a large selection of popular metrics.

## 1. Introduction

Automatic text simplification (ATS) aims at generating a text that is easier to read and understand while preserving the meaning of the original text (Saggion, 2017). Assessing whether a simplified sentence preserves the meaning of the original complex one is not trivial for a machine, and it is critical for correct simplification. It is crucial to many natural language processing (NLP) tasks, such as summarization, and machine translation.

Meaning preservation between sentences is one of the dimensions evaluated in natural language generation (NLG) tasks (Gatt and Krahmer, 2018). For example, ATS metrics evaluate three dimensions of system output generation: “fluency,” “simplicity,” and “meaning preservation.” Fluency measures grammatical correctness, simplicity measures how simple the output is, and meaning preservation measures how well the meaning of the output text corresponds to the meaning of the source (Saggion, 2017). Existing metrics such as BLEU (Papineni et al., 2002) and SARI (Xu et al., 2015) tend to focus on only one of the three dimensions, and none are designed explicitly for meaning preservation. BLEU is commonly used to cover fluency (Sulem et al., 2018a), while SARI (Xu et al., 2015) covers simplicity. Since none were designed for meaning preservation, they correlate poorly with human evaluation (Wubben et al., 2012; Alva-Manchego et al., 2020) and cannot be used to analyze complex text simplification tasks such as sentence splitting (Sulem et al., 2018a).

Today, most evaluation metrics used in NLP rely on progress made in language modeling research and use BERT-like models to compute the similarity between two sentences (Zhang et al., 2019; Zhao et al., 2019; Vasilyev et al., 2020; Scialom et al., 2021a; Maddela et al., 2022). For example, using contextualized word embeddings, BERTScore (Zhang et al., 2019), a metric that leverages large language models (LLM), computes the pairwise token-level similarity of two sentences. LENS (Maddela et al., 2022), a trainable evaluation metric for text simplification, also uses word embeddings and a ranking loss function focusing on the system output edit operations (e.g., splitting, deletion, and paraphrasing). However, these similarity metrics fail to evaluate the preservation of meaning between two sentences, which are an essential indicator of meaning preservation itself, as similar sentences tend to share similar meanings. For example, BERTScore correlates positively with human judgments on meaning preservation (Laban et al., 2020; Scialom et al., 2021a).

Correlation with human judgment is only one way to evaluate the quality of a meaning preservation metric. Alternatively, one can run benchmarking tests, such as evaluating meaning preservation between identical sentences (which should be 100% preserving) or unrelated sentences (which should be 0% preserving). Unfortunately, as we will show in this study, many of the above metrics fail even such simple tests.

In this study, we propose MeaningBERT^1^ (subsection 3.4), a new metric designed to assess meaning preservation between two sentences for text simplification, which we have trained to correlate with human judgment. To demonstrate its quality and versatility, we will also present, in our related work (section 2), a compilation of datasets used to assess meaning preservation using a continuous scale (0–100). In section 3, we will describe our experimental setup to compare our work against a large selection of metrics in the literature applied to these datasets and our sanity checks. Finally, we will analyze and discuss our results in section 4.

## 2. Related work

### 2.1. Human-evaluated text simplification datasets

Only a few text-simplification English datasets are available, and even fewer include human judgment on meaning preservation.

ASSET (Alva-Manchego et al., 2020) is a two-component dataset. The first component consists of 2,359 source sentences aligned with ten human-written simplifications for 23,590 source-human simplified sentence pairs. Using a continuous scale, the second component comprises 100 source-system simplified sentence pairs and 15 human evaluations per sentence on our three simplification dimensions, i.e. fluency, simplicity, and meaning preservation.

Simplicity-DA (Alva-Manchego et al., 2021) is a dataset comprising the simplification of 100 source sentences by six simplification systems. Each of the 600 simplified sentences was annotated by 15 humans using a continuous scale on all three simplification dimensions.

The SimpEval dataset (Maddela et al., 2022) is a four-component dataset. The first component, SimpEval_ASSET_, is a human-annotated dataset of 100 source sentences simplified by 24 automatic systems, for a total of 2,400 simplifications. Each simplification was evaluated by five annotators on their overall quality using a continuous scale for a total of 12,000 annotations. The second component, SimpEval_2022_, was created to address the possibility of “data contamination” within the ASSET component. This component includes 60 completely new source sentences, which were simplified by three human annotators and three different automatic systems, for a total of 360 simplification pairs. Then, three different human annotators evaluated simplification using the same approach as SimpEval_ASSET_. The last two components, SimpDA_2022_ and SimpLikert_2022_, are variations of the SimpEval_2022_ dataset. Both include 1,080 source-simplification pairs (360 sentences simplified three times). The first measures each transformed sentence's fluency, meaning and simplicity using a continuous scale, while the second uses a Likert scale (1–5).

The QuestEval dataset is an extended version of ASSET introduced by Scialom et al. (2021b). Each of the 100 source sentences of SimpEval_ASSET_ is simplified by a human annotator, and that simplification is evaluated by 30 human annotations using a continuous scale^2^ on all three simplification dimensions.

### 2.2. Human evaluation and automatic metrics for meaning preservation

Since automatic metrics serve as a proxy for human judgments on all three dimensions of text simplification, they should correlate well with human ratings.

Xu et al. (2015) were the first to study the correlation between automatic metrics and human judgments for text simplification. They found significant correlations between SARI and BLEU on fluency and meaning preservation. Their analysis was conducted on human-written and system-generated simplifications, however. As Scialom et al. (2021b) later pointed out, “given poor system performance at the time, significant correlations could be due to the very different quality of simplifications between systems and humans only, but not be able to differentiate different systems.” This realization actually motivated Scialom et al. (2021b) in creating their extension to the ASSET dataset.

On the other hand, Sulem et al. (2018a) found low to no correlation between BLEU and the fluency and meaning preservation dimensions when sentence splitting is applied. Also, they found BLEU often negatively correlates with simplicity, essentially penalizing simpler sentences.

Alva-Manchego et al. (2020) also analyzed the correlation with evaluation metrics of their newly-introduced ASSET dataset. Like Xu et al. (2015), they concluded that BLEU and SAIR correlate positively with meaning preservation and fluency. Furthermore, they also studied the correlation of human ratings with text features such as compression level and sentence length. Their results show that judgments on meaning preservation correlate with making few changes to the sentence.

As Gatt and Krahmer (2018) pointed out, various factors can be adduced to explain the inconsistencies between these meta-evaluations on the correlation of meaning preservation metrics and human judgment. Namely, BLEU is sensitive to the length of the texts being compared, a key element in Sulem et al. (2018a) argument on the inadequacy of BLEU for text simplification. Moreover, metrics such as BLEU do not consider semantic variability between original and transformed sentences that differ on synonymous words or in word order variations.

More recent metrics leverage recent progress in LLM, and thus can better account for semantic variability between the source and output text (Zhang et al., 2019; Brown et al., 2020). Given this, Scialom et al. (2021b) have conducted a correlation analysis on all three dimensions of text simplification for six metrics, including two that rely on LLM. They are the popular Flesch-Kincaid Grade Level (FKGL) metric (Kincaid et al., 1975), BLEU, SARI, BERTSCore and QuestEval, and their proposed approach. Their results show that BERTScore and QuestEval correlate more to human judgment on all three aspects of text simplification than BLEU and SARI.

Maddela et al. (2022), have conducted a similar analysis using LENS – a trained metric for text simplification quality assessment, have conducted a similar correlation analysis as Scialom et al. (2021b). Namely, they compare LENS against FKGL, BLEU, SARI, and BERTScore. Using a different dataset from the one used in Scialom et al. (2021b), their results also show that BERTScore largely improves over BLEU and SARI for correlation to human judgment on all three aspects of text simplification, and that LENS outperforms BERTScore on all three aspects.

## 3. Experimental setup

In this section, we discuss our experimental setup. First, we discuss the characteristics of the selected datasets in subsection 3.1. Then, in subsection 3.2, we present the 30 studied metrics in this work. We also made available in our Supplementary material more technical details about our experiments that are less relevant to the reader but are necessary for reproducibility. In subsection 3.3, we present our two sanity checks used in our experiments. Finally, in subsection 3.4, we discuss MeaningBERT training details.

### 3.1. Selected datasets

We use all four English datasets introduced in subsection 2.1 for our experiments. We named this merged dataset the “Continuous Scale Meaning Dataset.” Table 1 presents the number of sentence-simplification pairs in each of the four datasets, along with an example of such a pair and the average rating. We keep the datasets' average human-evaluated meaning preservation rating for each sentence pair.

**Table 1:** Dataset statistics and examples of the four datasets used for our experiment.

**Dataset**	**Number of sentence pair**	**Sentence pair example**		**Rating**
		**Source**	**Simplified**	
ASSET	100	It is not actually a true louse	It is not in fact a true louse	95.07
Simplicity-DA	600	He was appointed Companion of Honor (CH) in 1988	He was made a Companion of Honor in 1988	93.33
SimpDA_2022_	360	England are the reigning T20 World Cup holders, having beaten Pakistan in the 2022 final, winning their second title	The current T20 World Cup holder is England	32.00
QuestEval	295	There he had one daughter, later baptized as Mary Ann Fisher Power, to Ann (e) Power	There he had one daughter, baptized	51.07

To further analyze the corpora, Table 2 presents a quantitative textual analysis of the four corpora. The lexical richness (Van Hout and Vermeer, 2007) is the dataset ratio of unique words over its total number of words without removing the stop words. We can see that vocabulary size and sequence length, both token-wise and lexical (i.e., non-stopwords; LW), are relatively similar across all four datasets. However, SimpDA_2022_ has a vocabulary roughly two times larger than the other datasets, and Simplicity-DA has sentences about two times longer.

**Table 2:** Aggregate statistics on textual data of the four datasets used for our experiment.

	**ASSET**		**Simplicity-DA**		**SimpDA**_**2022**_		**QuestEval**	
	**Source**	**Simplification**	**Source**	**Simplification**	**Source**	**Simplification**	**Source**	**Simplification**
Vocabulary size	994	767	1,138	1,787	2,599	2,544	996	1,074
Vocabulary size lexical words (LW)	858	645	1,001	1,600	2,401	2,349	860	927
Avg sentence length (tokens)	18.25	12.98	38.45	20.83	19.75	15.92	18.91	14.00
Avg sentence length (LW)	9.94	6.72	20.74	11.00	10.96	8.44	10.37	7.44
Lexical richness	0.86	0.84	0.13	0.25	0.37	0.44	0.28	0.37

Furthermore, Table 3 presents the quartile distribution of all four datasets' meaning preservation ratings. We can see similarities between the datasets, notably having a similar mean, standard deviation and max rating. However, the table also shows notable differences, such as the Simplicity-DA minimum rating being greater than the first quartile of the ASSET dataset. Overall, we can see that the Simplicity-DA ratings have higher values than the others, and ASSET tends to have the lowest.

**Table 3:** Aggregate statistics on meaning preservation rating data using a continuous scale (0–100) for the four datasets used in our experiment.

	**ASSET**	**Simplicity-DA**	**SimpDA_2022_**	**QuestEval**	**Merged dataset**
Min	0.87	28.33	1.27	4.00	0.87
25%-quartile	21.37	82.67	43.23	52.57	52.20
Median	45.80	91.00	64.10	78.07	76.33
Mean	49.26	87.55	61.75	69.49	69.37
Standard deviation	30.57	12.98	23.77	24.93	25.33
75%-quartile	77.78	97.33	82.03	90.15	90.78
Max	99.47	100.00	98.67	99.47	100.00

For our experiment, we create a merged version of all corpus, since all four corpus share a similar definition of meaning preservation using a continuous scale (0–100) and have used a similar crowdsourcing annotation methodology based on Alva-Manchego et al. (2020, 2021), Scialom et al. (2021a), and Maddela et al. (2022). Alva-Manchego et al. (2020) data collection protocol requires participants (i.e., annotator) to have a HIT approval rate ≥95%, have a number of HITs approved >1, 000, are residents of the United States of America, the United Kingdom or Canada, and passed the corresponding “Qualification Test” designed for the task.

To create our dataset, we first assess if duplicates exist between the corpus designed to merge the datasets. To do so, we compare each triple (source, simplification sentence, and rating) against all the other tuples and reject duplicates. Duplicates were only found between the ASSET and QuestEval datasets.

Our dataset comprises 1,355 triplets: a source and simplified sentence pair with an average human-evaluated meaning preservation rating.

### 3.2. Selected metrics

Few works studied a broad range of metrics for textual simplicity (Alva-Manchego et al., 2021), and none focused on assessing meaning preservation between two sentences. Our study focuses on 21 automatic metrics divided into reference-based and non-reference-based approaches. The former are metrics that uses one or many human-annotated examples (i.e., references) to assess the quality of the first sentence compared to a second sentence. In contrast, the latter are metrics which do not require references to assess the meaning preservation between two sentences. Reference-based metrics can further be split into two approaches; metrics that use human reference sentences and a source sentence to assess a system dimension, or those that only use reference sentences. For example, BLEU is a reference-based metric that doesn't use a source sentence: it evaluates how close a system's output sentence is compared to a human-written reference sentence. Non-reference-based approaches are metrics that do not rely on human judgment to assess a system output. Most of these approaches try to quantify the improvement of a dimension of the task, such as simplicity improvement. For example, SAMSA (Sulem et al., 2018b) verifies the correctness of sentence splitting to quantify the structural simplicity of a system output sentence compared to the source sentence.

In total, 30 automatic metrics have been considered, from which 21 were selected for our studies based on two criteria. The first criterion, is that the metric must be used in either text generation, machine translation, or summarization tasks to assess the similarity between two sentences. And the second is that the metric must works on the newest Python version as of this publication (i.e., Python 3.11). This second criterion aims at minimizing potential conflicts between a metric and its use with state-of-the-art deep learning frameworks such as HuggingFace (Wolf et al., 2020). Since state-of-the-art solutions mostly rely on deep learning approaches and modern Python libraries, meaning preservation metrics must respect this practical constraint to be usable.

Below, we present each selected metric and provide a presentation of the metrics not selected in our Supplementary material.

#### 3.2.1. Reference-less metrics

**Flesch-Kincaid grade level (FKGL)** (Flesch, 1948) is a metric that computes the readability level of a piece of text. It does so by computing a weighted specific text ratio determined by well-documented research. It rates complexity level on a scale from 100 to 0, where 0 is the harder-to-read. This metric is mostly used in text simplification applications or any readability application due to its specific design.

#### 3.2.2. Reference-based metric

**BERTScore** (Zhang et al., 2019) uses contextual word embeddings and computes the cosine similarity between tokens of two sentences. The first sentence can be the system output simplified sentence, and the other is the human reference annotation. The metric provides three approach: BERTScore_Recall_, BERTScore_Precision_, and BERTScore_F1_. The first match each token in the reference sentence to its most similar in the system output, while the second matches the opposite (output to reference). The third combines the two like a typical equally weighted F1 score.
**BLANC** (Vasilyev et al., 2020) measure the quality of a summary by using a cloze-task on the source text to assess if a fine-tuned BERT model (i.e., a language model) can fill-in-the-blank masked word in the source text using the system output summary. The basic idea behind BLANC is that if a summary conveys the same information as the source text, thus it should help the BERT model unmask the masked token in the source text. To assess the summary contribution to the unmasking process, BLANC compares it with a baseline, namely unmasking the text without any summary.
**BLEU** (Papineni et al., 2002) is a well-know metrics in machine-translation. BLEU evaluates text quality by comparing a system output and a (professional) human-written reference. BLEU metrics count the matching words or n-grams of words between the two sentences. Following (Alva-Manchego et al., 2021) approach, we also experiment with the arithmetic mean (AM) and geometric mean (GM) of BLEU mixed with SAMSA, another metric introduced later.
**BLEURT** (Sellam et al., 2020) is a trainable metric for language generation built upon a BERT-like model. Part of the BLEURT training process uses machine translation data since it was developed for this kind of application. However, BLEURT is intended to evaluate text produced by language models.
**Coverage** was introduced by Laban et al. (2020) to assess the meaning preservation between a summary generated from a source text and the same source text masked. It is similar to the BLANC metric. Namely, it uses a cloze-task to assess if an LLM can fill in the blank using a summary generated from the same masked text. The main difference between BLANC and Coverage is that the latter accomplishes the cloze-task only with the summary and does not compare it with a reference value (i.e., fill in the blank without the help of the summary).
**iBLEU** (Xu et al., 2016) is the difference between a weighted combination using an α weight between the BLEU rating of the system output and the reference and of the source sentence and the reference. Namely, iBLEU does a normalization by removing the already overlapping words between the source sentence and the reference.
**FKBLEU** (Xu et al., 2016) is a metric that aims at leveraging both simplicity assessment efficiency of the text simplification FKGL metric and the meaning preservation capabilities (i.e., adequacy) of iBLEU. It combines the iBLEU metric of the system output sentence, the source sentence and the human reference with the FKGL difference between the system output and reference output simplicity ratings.
**LENS** (Maddela et al., 2022) is a metric for text simplification trained on the SimpEval_ASSET_ dataset introduce in subsection 2.1. It uses a pre-trained BERT-like architecture along with another learnable layer trained for text simplification quality assessment.
**METEOR** (Banerjee and Lavie, 2005) is a metric for machine translation evaluation using a generalized unigram concept matching between the system output sentence and human reference.
**QuestEval** (Scialom et al., 2019, 2021a; Rebuffel et al., 2021) is a metric designed to evaluate summarization, text simplification and data2text system output quality. The approach is built upon the SumEval (Fabbri et al., 2021) idea that uses an LLM to create a set of questions using a source sentence as a reference. Then it uses these questions and a question-answering model that uses simplified text to respond to them. If a simplified (or summarize) text conveys the same information as the source, a question-answering model should be able to respond properly to a set of questions based on the source text. The improvement of QuestEval is that it integrates a recall aspect to the SumEval framework to improve sentence correlation with human judgement.
**ROUGE** (Lin, 2004), a well-known metric in summarization and machine translation, measures the quality of a system output sentence compared to a human reference by matching the n-gram between the two sentences. It can compute a precision, recall and F1 rating of the matching n-grams and is typically calculated as a unigram length of 1, 2, or L for the longest common subsequence.
**SARI** (Xu et al., 2015) is a text simplification metric which compares a system output against a human-written reference and the source sentence. It measures the text simplification quality by measuring the goodness of words that are added, deleted and kept by the system.
**Sentence Transformer** is a framework for state-of-the-art sentence, text and image embeddings build upon Sentence-BERT (Reimers and Gurevych, 2019). It uses a siamese BERT-networks to compare two-sentence embeddings using an LLM. It can compute the cosine similarity between the two vectors.
**TER** (Olive, 2005) is a metric to quantify the edit operations that a system output sentence requires to match a reference translation. It generates a rating representing the number of edits compared to the human-written reference multiplied by 100.

We only selected one reference-less metric, even if other variants or approach similar to FKGL were introduced, such as the Gunning fog index (Gunning, 1969) and SMOG (Mc Laughlin, 1969). We did so since this metric is intended to measure simplicity and does not consider word use but instead word counts. Nevertheless, we choose to at least test the most well-use metric in text simplification as the bare minimum.

### 3.3. Sanity checks

Correlation to human judgment is one way to evaluate the quality of a meaning preservation metric. However, it is inherently subjective, since it uses human judgment as a gold standard, and expensive, since it requires a large dataset annotated by several humans. As an alternative, we designed two automated tests: evaluating meaning preservation between identical sentences (which should be 100% preserving) and between unrelated sentences (which should be 0% preserving). In these tests, the meaning preservation target value is not subjective and does not require human annotation to measure. They represent a trivial and minimal threshold a good automatic meaning preservation metric should be able to achieve. Namely, a metric should be minimally able to return a perfect score (i.e., 100%) if two identical sentences are compared and return a null score (i.e., 0%) if two sentences are completely unrelated.

#### 3.3.1. Identical sentences

The first test evaluates meaning preservation between identical sentences. To analyze the metrics' capabilities to pass this test, we count the number of times a metric rating was greater or equal to a threshold value *X*∈[95, 99] and divide it by the number of sentences to create a ratio of the number of times the metric gives the expected rating. To account for computer floating-point inaccuracy, we round the ratings to the nearest integer and do not use a threshold value of 100%.

#### 3.3.2. Unrelated sentences

Our second test evaluates meaning preservation between a source sentence and an unrelated sentence generated by a large language model.^3^ The idea is to verify that the metric finds a meaning preservation rating of 0 when given a completely irrelevant sentence mainly composed of irrelevant words (also known as word soup). Table 4 illustrated some of the sentences generated by the large language model to represent how far the two sentences are from each other. Since the expected rating is 0 in this test, we check that the metric rating is lower or equal to a threshold value *X*∈[5, 1]. Again, to account for computer floating-point inaccuracy, we round the ratings to the nearest integer and do not use a threshold value of 0%.

**Table 4:** Example of sentences with their unrelated (words soup) generated sentences using the procedure explained in our Supplementary material.

**Source**	**Irrelevant sentence**
One side of the armed conflicts is composed mainly of the Sudanese military and the Janjaweed, a Sudanese militia group recruited mostly from the Afro-Arab Abbala tribes of the northern Rizeigat region in Sudan.	Onstall larvaeauld Connell utility Lester give away enton Council engagement Khan batches lau.
Jeddah is the principal gateway to Mecca, Islam's holiest city, which able-bodied Muslims are required to visit at least once in their lifetime.	Definitions Volkswagen Spectrumongs communists 5 podcast Lakers migrate.
The Great Dark Spot is thought to represent a hole in the methane cloud deck of Neptune.	ost qualification condemns tice aston Sendrone Synt helm wiringHansnrossorup followed 222 Uncharted.

### 3.4. MeaningBERT

In addition, we propose MeaningBERT, the first supervised automatic metric of meaning preservation, which both correlates with human judgment and passes the sanity tests presented in subsection 3.3. MeaningBERT is built upon HuggingFace BERT-Base (uncased) model (Devlin et al., 2019), but uses a regression head instead of a classification one and fed sentences pair into the network by concatenating them with a [SEP] token. BERT-base is the smallest BERT model, with only 110 million parameters. We chose this model to reduce the model size and computation cost and thus to allow it to be used with other deep learning tasks.

To train MeaningBERT, we have used our merged dataset of section 4. In addition, to train MeaningBERT to pass our two sanity checks, we also generated a set of identical-sentence triplets (Sent_A_, Sent_A_, 100), and of unrelated-sentence triplets (Sent_A_, Sent_Unrelated_, 0) using the same procedure as the one used for the sanity checks. We thus have two training datasets, one comprising only 1,355 sentence triplets taken from our merged datasets and a second that augments the first with 2,710 sanity-check sentence triplets for 4,065 sentence triplets. We will refer to them as “Without DA” (e.g., Data Augmentation) and “With DA” respectively.

We have trained MeaningBERT by fine-tuning BERT using either “Without DA” or “With DA” dataset. Each model-dataset pair was trained using a 10-fold approach, using a different random seed to split the dataset and initialize the new regression attention head weights ([42, 43, ⋯ , 51]). The models were trained for 250 epochs with an initial learning rate of 5*e*^−5^, and we use a linear learning rate decay as suggested by Mosbach et al. (2021). The training dataset was divided using a 60%–10%–30% train-validation-test split with simple random sampling, resulting in 853 training samples. We use a batch size of 16 for training and 64 for evaluation. Training takes between 3 to 6 h, depending on the dataset used.

All selected metrics and MeaningBERT are evaluated on the same test split during the fold test phase. In addition, to benchmark all of our selected metrics and MeaningBERT on our two sanity checks, using completely unseen sentence (Sent_A_) taken from the test set of the ASSET corpus, we also generated a set of identical-sentence triplets (Sent_A_, Sent_A_, 100), and of unrelated-sentence triplets (Sent_A_, Sent_Unrelated_, 0). This sanity check dataset comprises 359 sentence-pair per sanity check (e.g., 718 in total).

## 4. Metrics ratings analysis

In this section, we analyze the selected metrics ratings for their ability to evaluate meaning preservation. We focus on two aspects for our analysis. First, we investigate how well each metric's evaluation of original-simplified sentence pairs correlates with human judgment. We also evaluate the metrics, as in a machine-learning model, using their rating as predictions of the human judgment using typical statistical regression evaluation metrics. Second, we create a set of “sanity checks” that metrics should be able to pass to be meaningful to human users, and apply them to each metric.

### 4.1. Correlation with human judgments

The first aspect of our analysis is to investigate how well each metric and MeaningBERT corresponds to the human judgment of meaning preservation. We first use Pearson correlation (Zar, 2005) to determine how well the values generated by each metric correlate with human annotations. Secondly, we evaluate the rating of each metric as if they were predictions of human judgment, using typical statistical regression evaluation metrics, namely *R*^2^ (James et al., 2013) and RMSE (James et al., 2013). This dual approach will give us a more complete quantitative view of the metrics' reliability as human approximators.

Table 5 presents the results of our 21 metrics and MeaningBERT for each of the three tests. In this table, bolded values are the best results per test.^4^ Finally, we recall that all selected metrics and MeaningBERT have been evaluated using a 10-fold approach using the same test set during the fold test phase; thus, the table displays the average score and a standard deviation.

**Table 5:** Results of the benchmarking metrics and MeaningBERT trained without data augmentation (DA) and with DA.

**Metrics**	**Pearson**	**R^2^**	**RMSE**
**BERTScore F1**	***0.426 ± 0.04***	**−0.946 ± 0.05**	**35.167 ± 0.82**
BERTScore precision	*0.424 ± 0.03*	−1.018 ± 0.05	35.813 ± 0.83
BERTScore recall	*0.391 ± 0.04*	−0.884 ± 0.04	34.596 ± 0.82
BLANC	0.1805 ± 0.03	−2.47 ± 0.03	47.15 ± 0.83
BLEU	*0.202 ± 0.03*	−1.56 ± 0.19	40.294 ± 0.81
BLEU-SARI (AM)	0.174 ± 0.03	−2.183 ± 0.21	44.935 ± 0.61
BLEU-SARI (GM)	0.169 ± 0.03	−2.385 ± 0.22	46.341 ± 0.61
BLEURT	*0.507 ± 0.05*	−5.338 ± 0.47	63.426 ± 2.47
Coverage	0.175 ± 0.06	−0.312 ± 0.08	28.877 ± 1.18
FKBLEU	0.097 ± 0.03	−6.251 ± 0.46	67.822 ± 0.79
FKGL	*0.209 ± 0.03*	−5.793 ± 0.42	65.647 ± 0.68
iBLEU	*0.202 ± 0.03*	−2.11 ± 0.22	44.407 ± 0.67
LENS	*0.529 ± 0.04*	−0.521 ± 0.11	31.066 ± 0.84
METEOR	0.189 ± 0.05	−0.328 ± 0.09	29.029 ± 0.86
QuestEval	*0.42 ± 0.04*	0.141 ± 0.04	23.352 ± 0.31
ROUGE-1	0.196 ± 0.05	−0.207 ± 0.06	27.691 ± 0.71
ROUGE-2	*0.203 ± 0.03*	-0.66 ± 0.1	32.451 ± 0.59
ROUGE-L	0.166 ± 0.04	−0.304 ± 0.05	28.789 ± 0.69
SARI	0.081 ± 0.04	−3.393 ± 0.28	52.79 ± 0.66
Sentence Transformer	*0.496 ± 0.02*	−0.289 ± 0.02	28.632 ± 0.94
TER	0.168 ± 0.03	−0.748 ± 0.09	33.313 ± 0.84
MeaningBERT (without DA)	0.251 ± 0.1	−0.009 ± 0.08	25.673 ± 0.89
MeaningBERT (with DA)	***0.928 ± 0.02***	**0.86 ± 0.03**	**16.355 ± 1.18**

Bolded values are the best results, and italic are results with a p-value α ≤ 0.999.

First, we can see that Pearson correlation scores varies greatly between metrics, with an average correlation of 0.2626 and a maximum of 0.5638 (LENS). From these results, we can reject the null hypothesis that the two samples of ratings and scores are independent for only a few metrics: BERTScore, BLEU, BLEURT, FKGL,iBLEU, LENS, QuestEval, ROUGE-2, Sentence Transformer and MeaningBERT with DA. It means that a statistically significant positive correlation exists between these metrics and human evaluation, although it is far from a perfect correlation.

Furthermore, it can be seen that metrics can be separated into two groups based on their Pearson score, namely those that achieve a correlation below 0.21 and those that achieve a correlation above 0.39. The first group comprises mostly n-grams overlapping metrics, while the second group is comprised only of newer metrics (BLEURT, LENS, Sentence Transformer, BERTScore, and MeaningBERT) that use a BERT-like model. It shows that relying on LLM semantic capabilities (Brown et al., 2020) correlates better with human judgment for meaning preservation. However, these metrics have been trained using large text corpora collected from online resources and thus, as pointed out by Maddela et al. (2022), parts of our dataset might be part of their training corpus. However, none have been trained to do meaning preservation assessments using the ratings we computed, with the exception of LENS which has been trained on SimpDA_2022_ (nearly 27% of our dataset). This might explain why LENS achieves a so much stronger correlation value than the other LLM metrics.

On the other hand, we can see that all the metrics achieve poor performance on the two machine learning evaluations, with only two metrics achieving a positive *R*^2^ (QuestEval and MeaningBERT with DA). The most correlated metrics tend to perform poorly on the *R*^2^ score. It shows that predicting relative quality according to human judgements and predicting human evaluation are two different challenges. Some metrics put the pairs in the same order as humans without being able to find the correct values (e.g., BLUE), and we have metrics that come closer to the actual values of human judgments, but the error goes all over the place, so the relative order is lost (e.g., ROUGE-L).

Finally, we can see that MeaningBERT with DA outperforms all other metrics on all three evaluation metrics by a wide margin. It achieves nearly twice the LENS Pearson correlation score (second best) while having half the RMSE score. Moreover, it is the only metric to achieve a positive *R*^2^. Also, our data augmentation technique improves performance on all aspects of our evaluation. It indicates that proper data augmentation can significantly increase performance.

### 4.2. Metrics sanity checks

This section investigates how well each metric and MeaningBERT pass our two sanity checks.

#### 4.2.1. Relevant sentence results

The results of the first sanity check are presented in Table 6, bolded values are the best results per column. First, We can see that most metrics always return the near-expected value of 100% (e.g., 99% to account for rounding error) when comparing two identical sentences: SARI, BLEU, METOR, TER, ROUGE, BERTScore, Sentence Transformer and MeaningBERT (and their respective variations). These results are expected for the five first metrics since all these approaches rely on n-grams overlap between the two sentences. Thus, two similar sentences necessarily have a near-perfect match. On the other hand, BERTScore and Sentence Transformer sanity check pass is due to the FACT that both approaches that compare sentence embeddings; the first with a token-wise match and the second using the cosine similarity, resulting in two identical sentence embeddings.

**Table 6:** Percentages of time a metric returns the expected rating for the unrelated sentence test using the sanity check dataset same sentence split.

**Metrics**	**% greater than 95%**	**% greater than 99%**
BERTScore F1	**100 ± 0.0**	**100 ± 0.0**
BERTScore precision	**100 ± 0.0**	**100 ± 0.0**
BERTScore recall	**100 ± 0.0**	**100 ± 0.0**
BLANC	3.36 ± 0.0	3.36 ± 0.0
BLEU	**100 ± 0.0**	**100 ± 0.0**
BLEU-SARI (AM)	**100 ± 0.0**	**100 ± 0.0**
BLEU-SARI (GM)	**100 ± 0.0**	**100 ± 0.0**
BLEURT	8.914 ± 0.0	2.228 ± 0.0
Coverage	35.655 ± 0.0	0.279 ± 0.0
FKBLEU	0 ± 0.0	0 ± 0.0
iBLEU	0 ± 0.0	0 ± 0.0
LENS	0 ± 0.0	0 ± 0.0
METEOR	**100 ± 0.0**	**100 ± 0.0**
QuestEval	3.064 ± 0.0	0.557 ± 0.0
ROUGE-1	**100 ± 0.0**	**100 ± 0.0**
ROUGE-2	**100 ± 0.0**	**100 ± 0.0**
ROUGE-L	**100 ± 0.0**	**100 ± 0.0**
SARI	**100 ± 0.0**	**100 ± 0.0**
Sentence Transformer	**100 ± 0.0**	**100 ± 0.0**
TER	**100 ± 0.0**	**100 ± 0.0**
MeaningBERT (without DA)	0 ± 0.0	0 ± 0.0
MeaningBERT (with DA)	**100 ± 0.0**	**100 ± 0.0**

Bolded values are the best results.

Second, Coverage and BLANC perform poorly on the test, especially given their approach. Both rely on a cloze-task: using an LLM to unmask the source sentence using the simplified sentence as a context. Thus, unmasking a masked sentence using the original sentence should be easy. This poor performance is due to how ratings are computed. Instead of computing the number of properly unmasked words over the number of masked words, they compute the sum of the probability of properly unmasked words over the number of masked words (Laban et al., 2020; Vasilyev et al., 2020). Thus, if the probabilities of the properly unmasked words are not all equal to nearly 100%, the score cannot be close to the expected behavior.

Finally, we can see that approaches that use a regression head (LENS, BLEURT, and MeaningBERT without DA) to assess two sentences' similarities perform poorly compared to similar approaches that use DA. It shows that without a proper corpus that includes such sanity checks example used during training, BERT approaches tend to be too pessimistic and never return ratings equal to 100% even if the two sentences are identical.

#### 4.2.2. Irrelevant sentence results

The results of this test are presented in Table 7, bolded values are the best results. The most striking observation is that, unlike for the previous test, only four metrics achieve a perfect performance. However, we can see that the rating values are relatively low, with most of these metrics returning ratings below 5%, and few can generate scores close to 1% nearly all the time. For SARI, BLEU, TER, and ROUGE (and their variations), this situation is probably due to the fact that they use n-grams comparisons. Even when compared to an unrelated word soup, some overlap with the original sentence can occur, resulting in a non-zero rating. For the BERT-like metrics, we hypothesize that contextualized embeddings and the underlying LLM make it possible to hallucinate connections and common meaning between the two sentences even when none exists, thus returning a non-zero rating.

**Table 7:** Percentages of time a metric returns the expected rating for the unrelated sentence test using the sanity check dataset unrelated sentence split.

**Metrics**	**% lower than 5%**	**% lower than 1%**
BERTScore F1	0 ± 0.0	0 ± 0.0
BERTScore precision	0 ± 0.0	0 ± 0.0
BERTScore recall	0 ± 0.0	0 ± 0.0
BLANC	83.03 ± 1.0	78.60 ± 0.85
BLEU	**100 ± 0.0**	71.253 ± 2.39
BLEU-SARI (AM)	**100 ± 0.0**	69.415 ± 2.67
BLEU-SARI (GM)	**100 ± 0.0**	69.916 ± 2.79
BLEURT	**100 ± 0.0**	**100 ± 0.0**
Coverage	6.017 ± 1.05	1.114 ± 0.52
FKBLEU	**100 ± 0.0**	91.198 ± 1.98
iBLEU	**100 ± 0.0**	91.142 ± 1.92
LENS	**100 ± 0.0**	99.387 ± 0.36
METEOR	97.103 ± 1.32	61.838 ± 1.51
QuestEval	0 ± 0.0	0 ± 0.0
ROUGE-1	**100 ± 0.0**	94.986 ± 0.86
ROUGE-2	**100 ± 0.0**	**100 ± 0.0**
ROUGE-L	**100 ± 0.0**	94.986 ± 0.86
SARI	**100 ± 0.0**	68.635 ± 2.96
Sentence Transformer	52.925 ± 1.21	28.022 ± 1.71
TER	**100 ± 0.0**	**100 ± 0.0**
MeaningBERT (without DA)	0 ± 0.0	0 ± 0.0
MeaningBERT (with DA)	**100 ± 0.0**	**100 ± 0.0**

Bolded values are the best results.

## 5. Conclusion and future work

This paper proposed a new metric to assess meaning preservation between two sentences, specifically in the context of text simplification, although our metric could be used for other tasks as well. To demonstrate its quality and versatility, we also presented a compilation of datasets used to assess meaning preservation and compared our work against a large selection of popular metrics in the literature applied to these datasets. Further, we introduced two automatic sanity checks for meaning preservation: evaluating meaning preservation between identical and unrelated sentences and evaluating our method and the benchmark metrics in these tests. In future work, we aim to study how MeaningBERT generalizes on other languages and tasks.

## Data availability statement

The dataset, “Continuous Scale Meaning Dataset”, used for this study can be found in the official GitHub repository: https://github.com/GRAAL-Research/csmd.

## Author contributions

DB, HS, and RK contributed to the conception and design of the study. DB performed the experimental setups, code development, statistical analysis, and wrote the first draft of the manuscript. All authors contributed to the manuscript's revision, read, and approved the submitted version.

## Footnotes

^1^ https://github.com/GRAAL-Research/MeaningBERT

^2^We found several discrepancies between the paper's method and the official codebase implementation by the authors. We list our findings in our Supplementary material.

^3^See our Supplementary material for more details on how we generated those sentences.

^4^To mitigate confusion between automatic metrics “score” studied in this work (e.g., SARI, BLEU) and automatic metrics “score” used to evaluate these metrics (e.g., Pearson), we will refer to the former as “ratings” and the latter as “scores.”
