# Supplementary material for: MeaningBERT: assessing meaning preservation between sentences
Source: Front Artif Intell. 2023 Sep 22;6:1223924. doi: 10.3389/frai.2023.1223924 (PMC10557945; doi:10.3389/frai.2023.1223924)
Supplement: Supplementary file 1 [file Data_Sheet_1.PDF]

Supplementary Material

1 QUESTEVAL DISCREPANCIES DISCUSSIONS

As highlighted in our article, we found several discrepancies between the paper's method and the official codebase implementation by the authors. Here we list our observations.

First, the authors stated in their article that they use a liker five-level scale, but their data instead suggest a continuous scale.

Second, they state that ASSERT includes 9,000 annotated system simplifications, while it is 4,500. Moreover, based on their online dataset, the dataset seems to comprise 14,823 annotations. 9,357 are "human" generated simplification (authors contribution) and 5,466 from systems (taken from ASSET second component dataset). We manually and programmatically investigated the dataset to assess if it included duplicates using the source and simplification sentence as merging key, leading to 8,850 annotations.

Finally, based on the released dataset, it includes 295 new sentence-simplified pairs written by humans (as opposed to the system generated in the ASSET dataset), each having 30 human evaluations on all three dimensions, so a total of 8,850 annotations for each dimension.

2 METRICS NOT SELECTED

In total, nine automatic metrics have been considered but were not selected based on the same three criteria discussed in the selected metrics section. Documenting and reporting on all metrics would take much work, and since it is not our main objective, we focus on well-known, well-used, or closely related metrics that offer promising interest for our work. Like the previous section, we will present and discuss each metric and its specific use area.

2.1 Reference-Based Metric

MAUVE (Pillutla et al., 2021) measure the gap between two sets of text. It is computed using the Kullback–Leibler (KL) divergences between the two text distributions in a quantized embedding space of a large language model. It does so by measuring, using a k-means algorithm and text embedding, the area under a divergent curve between two sets of text. MAUVE correlates well with a different dimension of text quality, such as the human-like look of system output text and text interest. The metric is designed for open-ended text generation (i.e. dialogue generation). However, to work properly, MAUVE documentation suggests using at least 1,000 samples of each text distribution to create relevant results. Since we compare two sentences, MAUVE is not an appropriate metric for our study. For this reason, MAUVE was not selected for our experiments.

MoverScore (Zhao et al., 2019) use contextualized embeddings to compute the Euclidean distance between words or n-grams. In contrast to BERTScore, which only allows one-to-one hard matching of words, MoverScore allows many-to-one matching since it uses a soft partial alignment similar to the Word Mover's Distance (WMD) (introduced later). Like BERTScore, MoverScore can be used in many applications such as summarization (Jin et al., 2020). However, the approach required a TF-IDF matrix on all the corpus to work properly; thus, single-only use is useless. Official Python documentation suggests there is a sentence level without the necessity of a TF-IDF matrix, but it is not included in the codebase. Moreover, the current implementation uses an outdated version of the

HuggingFace Transformers library[1], raising concern about its maintainability for future uses. Finally, the documentation lacks proper explication on using and setting MoverScore, making it more difficult to use and understand properly. For all those reasons and limitations, and since BERTScore uses a similar approach, we choose not to select MoverScore.

ORANGE (Lin and Och, 2004) is a metric development for machine translation aiming at reducing the quantity of human involvement. It uses ranked reference traduction as a set of n-best alternative translations given a source sentence using traduction systems (i.e. an oracle system). It then computes the ratings for the n-best source-reference traduction, ranks them and computes a rating using the average of all the ranked pairs. Such an approach could be interesting for text simplification since it uses a set of acceptable human-written examples rather than focusing on a single reference and uses it to rank the system output. However, since it uses a machine translation system as the oracle, rating quality might not be as relevant for an out-of-the-box use for text simplification. Moreover, we could not find any reference to an official implementation either in the article or using a Web search. For these two reasons, ORANGE was not selected for our experiments.

SUPERT (Gao et al., 2020) metrics rate neural generative system summary by measuring its semantic similarity with pseudo reference summary. The pseudo reference summary is generated using salient sentence extraction from the source document using contextualized embedding and a soft word token alignment. Thus, the metric is inappropriate since we work at a sentence level. For this reason, we did not select SUPERT.

The **Word Mover-Distance (WMD)** (Kusner et al., 2015) is a metric introduced to measure dissimilarity between text documents by computing the minimum cumulative distance between the embeddings of their constituent word. It uses an optimal matching between word embeddings based on the Euclidian distance instead of a classical geometrical mean used to average word embeddings to create sentence or document embeddings. This approach is designed for documents rather than sentences. Thus, it is not suited for our experiments. For this reason, we choose not to select this metric. However, the approach could be a promising approach to adapt on the sentence level for text simplification and meaning preservation since it could map word embeddings between two texts and thus be more robust to sentence splitting or paraphrasing.

2.2 Reference-Less Metric

SAMSA (Sulem et al., 2018) is a metric that focuses on automatic text simplification for sentence splitting assessment. It leverages semantic parsing to assess simplification quality. Thus, it is reference-less by decomposing the source sentence and comparing the decomposed system output sentence. Following (Alva-Manchego et al., 2021) approach, and similarly to BLEU and SARI, we also considered the arithmetic mean (AM) and geometric mean (GM) of SARI mixed with SAMSA. However, a major problem with SAMSA is that it takes much time due to its heavy use of semantic parsing. Based on SAMSA documentation, processing 120 sentences takes 4 minutes (see here). So, for a dataset like us (1,355 sentences pair) take roughly 45 minutes to process[2]. Making it difficult to use in the training process. Moreover, SAMSA uses outdated and conflicted dependencies (see here), making it difficult to install and raise concerns for maintainability and future use. For this reason, we choose not to select SAMSA for our experimentation.

[1] See here for more details.

[2] It is also worth mentioning that during our experimentation, we used more than 1,355 sentences, and we observed a longer processing time since we do not batch sentences due to our experiment approach.

3 TECHNICAL IMPLEMENTATION DETAILS

In this supplementary detail, we share technical details about our code to create a more reproducible article. First, we share the technical aspect of metrics implementations used for our experimentation. Second, we share some technical considerations to compute the rating, such as what we used as a reference and source when computing some metrics. Finally, we explain our methodology to generate the unrelated sentence used in our experimentation and training procedure for our data augmentation dataset.

3.1 Metric Technical and Implementation Details

3.1.1 Selected Reference-Based Approach

BERTScore is a well-maintained metric hosted on HuggingFace BERTScore Metric Hub Page and supported on Python version greater than 3.6. We have used the HuggingFace version of BERTScore for our experiment. As suggested by Zhang et al. (2019), we report the BERTScore metric hash use for our experiments: `4907b18f8f741f202232c0f8009a3bd-49ff98802c245abcb6ea51a37a8c05b`.

BLANC is relatively well maintained with an official implementation. It supports Python versions greater than 3.5. We have used the official Python implementation with the default configuration.

BLEU is a well-maintained metric with an "official" implementation hosted on HuggingFace BLEU Metric Hub Page[3] and supported on Python version greater than 3.5. We used the HuggingFace version of BERTScore for our experiment using the default parameters and English model (i.e. `roberta-large`).

BLEURT is hosted on HuggingFace BLEURT Metric Hub Page and support recent Python version. We have used the HuggingFace version of BERTScore for our experiment with the `bleurt-large-512` model. Like BERTScore, we report BLEURT model hash use for our experiments: `98e148b2f8c4a88aba5037e4e0e90c9fd9ec35dc37a054ded8cfef0fa801ffab`.

Coverage approach does not have an official implementation; thus we use the authors' official GitHub repository and extract the code for our experiment. Our implementation is available with our official repository GitHub repository[4]. We have used the same configuration as the authors.

iBLEU has no official implementation, but the algorithm is easy to implement since it relies on the BLEU metric. Our implementation is available with our official repository GitHub repository. We have used $\alpha = 0.9$.

FKBLEU has no official implementations, but the algorithm is easy to implement since it relies mostly on already implemented algorithms (i.e. BLEU and FKGL). Our implementation is available with our official repository GitHub repository. We have used $\alpha = 0.9$.

LENS' official implementation has conflicted dependencies in the installation process, making it difficult to install it. However, it can be fixed by removing some specified versions in the installation script. Thus, we forked the authors' official GitHub repository and fixed the dependencies conflicts. We have used our installable version of LENS available here[5]. We have used the authors' trained model available here.

[3] It use the SacreBLEU Python implementation.

[4] https://github.com/GRAAL-Research/MeaningBERT-article

[5] https://github.com/davebulaval/LENS

METEOR is hosted on HuggingFace METEOR Metric Hub Page and support recent Python version. We have used the HuggingFace version of METEOR for our experiment.

QuestEval official' implementation is relatively well maintained. However, similar to LENS, some dependencies are outdated, creating conflict. To this end, we use our installable version forked from the official GitHub repository available here[6].

ROUGE first version was written in Perl and is difficult to work with in Python. However, many ROUGE implementations have been proposed in Python over the year. One of the most known implementations is the one rewritten by Google Research also available on HuggingFace Metric ROUGE Hub. We have used the HuggingFace version of ROUGE for our experiment.

SARI is hosted on HuggingFace Metric METEOR Hub and support recent Python version. We have used the HuggingFace version of SARI for our experiment.

Sentence Transformer official's implementation is the SentenceTransformers Python library that supports Python version greater than 3.5. It is well-maintained and easy to use. We use this library in our experiment. We have used the third-best model base on the documentation performance using the sentence embeddings score as a selection ranking. We selected the third, `all-roberta-large-v1`, since the first two (`sentence-t5-xxl` and `gtr-t5-xxl` (in order)) are roughly 9 GO, which was a limiting factor given our available GPU of 12 GO (given the batch size and another metric computer in the same time). The performance difference between the best model and the third is 1.35 points.

TER is hosted on HuggingFace Metric TER Hub and support recent Python version. We have used the HuggingFace version of TER for our experiment.

3.1.2 Selected Reference-Less Approach

FKGL most known Python library that offers a proper FKGL implementation is Textstat. It is a well-maintained Python version, and it is easy to use.

3.1.3 Evaluation Metric Details

To compute our various evaluation scores, we have used the following libraries:

- For the **RMSE** evaluation metric we have use the Scikit-Learn' implementation.
- For the **Pearson correlation** evaluation metric, we have used the HuggingFace version of Pearson correlation.
- For the $\mathbf{R}^2$ evaluation metric, we have used the HuggingFace' implementation.

3.2 Code Implementation Details

In this section, we detail two technical choices we made in order to be able to compute some selected metrics. First, four metrics are computed using a set of references, a source and a system output: SARI, iBLEU, FKBLEU and LENS. Since we do not have a set of references (other than the simplified sentence), we chose to use the source as the set of references and the source. For all other metrics, the source is used only as a reference.

[6] https://github.com/davebulaval/QuestEval

4 GENERATION OF UNRELATED SENTENCE

In order to highlight the test case that a metric should be able to return a zero rating when two sentences are unrelated, we have used a large language model to generate these unrelated sentences; namely, we have used the GPT2-medium model (Radford et al., 2019) hosted on HuggingFace. Our generation procedure is inspired by other work that uses a large language model as a data augmentation generator (Kumar et al., 2020; Feng et al., 2020; Bayer et al., 2022). Our resulting procedure is as follows: We first ask the model to generate eight sentences using the source sentence as input. The model can generate a sentence of at most 90 tokens (`max_output_length`) and cannot generate more than five repeating n-grams. To increase the sentence's randomness and originality, we set a high-temperature value of 10. Finally, upon generation, we sort the sentence base on the generation score and take the lowest one as the system output to be used as the unrelated sentence.

REFERENCES

Alva-Manchego, F., Scarton, C., and Specia, L. (2021). The (Un)Suitability of Automatic Evaluation Metrics for Text Simplification. *Computational Linguistics* 47, 861–889

Bayer, M., Kaufhold, M.-A., and Reuter, C. (2022). A Survey on Data Augmentation for Text Classification. *ACM Computing Surveys* 55, 1–39

Feng, S. Y., Gangal, V., Kang, D., Mitamura, T., and Hovy, E. (2020). GenAug: Data Augmentation for Finetuning Text Generators. In *Proceedings of Deep Learning Inside Out: The First Workshop on Knowledge Extraction and Integration for Deep Learning Architectures*. 29–42

Gao, Y., Zhao, W., and Eger, S. (2020). SUPERT: Towards New Frontiers in Unsupervised Evaluation Metrics for Multi-Document Summarization. In *Proceedings of the Annual Meeting of the Association for Computational Linguistics*. 1347–1354

Jin, H., Wang, T., and Wan, X. (2020). samSUM: Semantic Dependency Guided Neural Abstractive Summarization. In *Proceedings of the AAAI conference on artificial intelligence*. vol. 34, 8026–8033

Kumar, V., Choudhary, A., and Cho, E. (2020). Data Augmentation Using Pre-trained Transformer Models. In *Proceedings of the on Life-long Learning for Spoken Language Systems Workshop*. 18–26

Kusner, M., Sun, Y., Kolkin, N., and Weinberger, K. (2015). From Word Embeddings To Document Distances. In *Proceedings of the International Conference on Machine Learning*, eds. F. Bach and D. Blei (Lille, France: PMLR), vol. 37 of *Proceedings of Machine Learning Research*, 957–966

Lin, C.-Y. and Och, F. J. (2004). ORANGE: A Method for Evaluating Automatic Evaluation Metrics for Machine Translation. In *COLING: Proceedings of the International Conference on Computational Linguistics*. 501–507

Pillutla, K., Swayamdipta, S., Zellers, R., Thickstun, J., Welleck, S., Choi, Y., et al. (2021). MAUVE: Measuring the Gap Between Neural Text and Human Text Using Divergence Frontiers. *Advances in Neural Information Processing Systems* 34, 4816–4828

Radford, A., Wu, J., Child, R., Luan, D., Amodei, D., Sutskever, I., et al. (2019). Language Models Are Unsupervised Multitask Learners. *OpenAI blog* 1, 9

Sulem, E., Abend, O., and Rappoport, A. (2018). Semantic Structural Evaluation for Text Simplification. In *Proceedings of the Conference of the North American Chapter of the Association for Computational Linguistics: Human Language Technologies*. 685–696

Zhang, T., Kishore, V., Wu, F., Weinberger, K. Q., and Artzi, Y. (2019). BERTScore: Evaluating Text Generation With BERT. In *International Conference on Learning Representations*

Zhao, W., Peyrard, M., Liu, F., Gao, Y., Meyer, C. M., and Eger, S. (2019). MoverScore: Text Generation Evaluating with Contextualized Embeddings and Earth Mover Distance. In *Proceedings of the Conference on Empirical Methods in Natural Language Processing*. 563–578
